# An Evaluation of Patient Satisfaction with Nursing Care: A Qualitative Study in an Indonesian Hospital

**DOI:** 10.4314/ejhs.v30i6.20

**Published:** 2020-11

**Authors:** Wardah, Said Usman, Elly Wardani

**Affiliations:** 1 Master Program of Nursing Science, University of Syiah Kuala, Banda Aceh, Indonesia; 1 Nursing Department, Sultan Abdul Aziz General Hospital, East Aceh, Indonesia; 2 Associate Professor, Faculty of Medicine, University of Syiah Kuala, Banda Aceh, Indonesia; 3 Assistant Professor, Faculty of Nursing, University of Syiah Kuala, Banda Aceh, Indonesia

**Keywords:** Culture, hospital management, Indonesia, nursing services, nurse, patient satisfaction, religious aspect

## Abstract

**Background:**

Patients experience first-hand quality services from nurses who are directly responsible for their welbeing. However, patient dissatisfaction with nursing services remains a problem in most developing countries. Therefore, this study aims to explore patient satisfaction with nursing care services in an Indonesian hospital.

**Method:**

A qualitative study with a descriptive phenomenology method was employed. Also, in-depth interviews were conducted with 15 informants, and thematic analysis was adopted to analyze the data.

**Results:**

The results of this study are described in the following themes and sub-themes: (1) hospital existence in public eyes: a) service commitment b) accessibility; (2) patients' background: a) religious aspect, b) cultural influence on perceiving health and sickness.

**Conclusion:**

Hospital management needs to enhance the quality of nursing services through sustainable education programs and continuous training. These are important to improve nurses' cognition and skills, and further to ensure patient satisfaction and hospital quality.

## Introduction

It is the essential responsibility of all health care providers to treat patients with the best facilities (1), because the quality and adequacy of medical service is measured on the basis of patient and family satisfaction and perspective (2). Therefore, patient satisfaction is the main indicator and evidence for standard service (3–6) and it is pertinent to be explored since it offers prominent details on the performance of health providers' and the quality of hospitals' management (7,8).

Patient satisfaction is one of the factors that determine service quality in hospitals, especially nursing care, which involves direct service delivery to patients. Therefore, the success of health services is solely dependent on the performance of nurses. Consequently, patient satisfaction is determined by the quality of nursing services. Furthermore, nurses spend most of their time with patients. Therefore, they play an important role in their overall satisfaction (9).

However, patient dissatisfaction on nursing services is a problem both in the developed and developing countries. A survey in Europe and the United States which involved 11,318 and 120,000 patients, respectively, showed a low percentage of the quality of nursing services; this ranges from 11% (Ireland) – 47% (Greece).

The National Health Insurance (JKN) is one of the several efforts made by the government to improve public health status and patients satisfaction. A previous study showed that JKN increases the service quality to patients with low economic status. However, some of them were not satisfied (10). Furthermore, an effort by the government to ensure adequate services was the establishment of standard accreditation for all hospitals in 2012, based on the regulation by Indonesian Ministry of Health (No.02).

Kusbaryanto stated that one of the importance of accreditation is the provision of standardized facilities and infrastructures to ensure hospital quality and patients' satisfaction (11). These efforts have been in place; however, complaints and dissatisfaction are reported on the care provided by the existing nurses (12). This study was, therefore, conducted to explore patients' satisfaction and experience with nursing care and to identify the factors that contribute to their contentment during hospitalization.

## Methods

This study adopted a qualitative design with phenomenology approach. Ethical approval and study permission were obtained from the University Research Ethics Committee and the director of the general hospital. A total of 15 respondents were randomly selected with several criteria, which include 3 or more days of hospitalisation, adult patients with stable condition, good consciousness, and the ability to communicate. Also, both oral and written informed consents were obtained and the participants were aware of their voluntary participation and their right to withdraw from the study at any time. Furthermore, the interview was conducted from 2^nd^ October to 12^th^ November 2019 in a General Hospital. Prior to this interview, each respondent was asked to fill demographic data including his/her occupation, education, length of stay, age, ethnicity and religion. The interviewer's guide was based on the dimension of service quality such as responsiveness, reliability, assurance and empathy during their time with the nurses.

This study uses thematic analysis (13) in five steps. This including understanding the data, initial coding, looking for and re-examining the theme, and finalized with writing the results and discussion. The main interviewer was the principal author who was capable and well-versed in both Bahasa Indonesia and Achenese language, and the other two supervised the research process and were vividly involved in ensuring the rigor of the study.

## Results

**Demographic data**: From a total of 15 Muslim respondents, 9(60%) were females while 6(40%) were males. Also, 8(53.3%) were hospitalized for more than 3 days, and 7(46.7%) were over 47 years old. Furthermore, 6(40%) of the participants had high school and the majority were housewives.

## Theme

**Figure 1:**
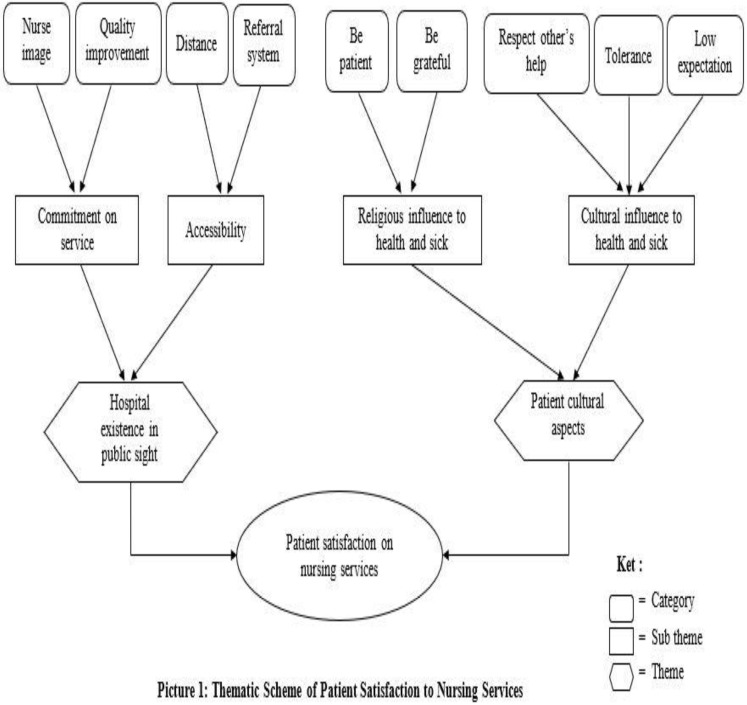
Thematic Scheme of patient satisfaction to nursing services

The categories identified in this study are shown in Figure 1. Each theme and the sub-theme are explained below.

**Hospital existence on public sight**: According to the participants, hospital existence on public sight was seen in hospital commitment. Most of them argued that the image of nurses was quite good. This is proved in their attitude and behaviour in the dispensation of medicine as well as their great knowledge and competencies when providing care.

“Nurses tell us about our health condition since we don't have enough knowledge. Also, they make instant provision for our needs, which is very nice…” (P1).

Some of the participants stated that good manner shown by nurses during their service delivery to patients tends to cure their diseases.

“Sitting here has not bothered our heart at all… I am always glad to see the nurses. We feel that our health has improved due to the special care of the nurses, even though the illness still exists in our body…” (P5).

The ninth participant also said:

“The nurses have good manner, they address us politely and treat us gently. They never frown, that is nice. We will get well…” (P9).

They also stated that there was an improvement in nurses' manner and responsiveness compared to the old time.

“I saw that the nurses are more friendly. It was quite different from before. Long ago, when we seek for their attention, we are not immediately attended to; we are not really cared for… Nowadays, they respond instantly to our call. The main thing is, their services are already better and they are friendly” (P8).

Others reported that accessibility also influences hospital existence in public sight. Besides the proximity of their residences, they are members of National Social Insurance (BPJS). Therefore, it is relatively easy to reach the hospital.

“We live in nearby villages., It is closer here. We are also under the national insurance” (P8).

**Patient's cultural aspect**: Religion influences welbeing and the state of health that emerges from the values, norms and behavioural beliefs of the patients. These, therefore, affect their perspective of nursing services. Furthermore, they believed that sickness is a test from the Almighty Allah, and whoever is afflicted will recieve forgiveness of sins as long as they exercise patience with the condition.

“… as for we getting sick, perhaps we already have many sins to Allah; by this Allah may forgive us” (P3).

The ninth respondent also said that sickness is a test and it is necessary to get Allah's forgiveness, as expressed below:

“We are sick. This is the test from Allah. In this way, may our sins be forgiven, we just need to be patient (P13)”.

Besides patience, the respondents also said that they must show gratitude to Allah, as expressed below:

“Everything that is given by Allah are for our wellbeing. Therefore, being grateful is better than complaining” (P12).

Cultural aspects from the patients also influence participants' views concerning nursing services. Muslim values as depicted above have a vivid impact on the way they see health and sickness. Furthermore, participants respect nurses' performance and assume that all services they recieve are satisfactory. Therefore, they need to be thankful.

*“I am really touched with their care* [showed sad expression and watery eyes]. *I always appreciate them when they administer medicine. I am grateful and fortunate to be here and we are indebted to them [*replied while wiping her tears]*”(P12).*

**Feeling of accepting the provided services**: Mistakes or shortcomings by the nurses is not a burden to very tolerant patients. This is evident in a patient's statement below:

“…We just want to be treated, irrespective of the service quality… it is enough. As I mentioned earlier, we have no complaints about them. Though they may have small flaws, it makes sense because they are human beings” (P8).

Furthermore, the interview with the participants showed that their expectation in nursing services is low. This is evident from the stated sentence of some participants below:

“…it was appropriate with our expectation, we should not expect very high” (P1).

*“I don't expect many things. I think they treated us well; their services are satisfactory. Alhamdulillah* (Praise to Allah) *(P15).*

## Discussion

The service quality of a hospital has positive and significant effects on customer satisfaction. A higher service quality significantly increases customer satisfaction and vice versa (14). Nursing services are an integral part of the hospital, a success indicator of its goals, and also a determinant of its image in public view. This, in particular, is due to the intense interaction between nurses and patients (15).

The quality of health services basically depends on patients' satisfaction with nursing activities, a high service quality amounts to patients satisfaction and vice versa (16). This is in line with Cao (17), who reported that caring attitude is the core of nursing services and the basic factor that establishes the relationship between patients and nurses (18). Furthermore, the manners, behaviours and communication skills of the hospital workers create the image before patients and their families. Even though the patients' expectations are not met, they usually feel quite satisfied that they were treated with respect for their feeling and prestige (19). This is consistent with previous research by Zeithaml who showed that perceptions of product quality affect its value (20).

Furthermore, all the participants are members of National Social Insurance (BPJS) that pay for all their treatment. With this insurance, it is expected that better healthcare will be provided from start to finish (10). In addition, the distance and referral system from the first healthcare provider significantly influences patient satisfaction to nursing services (21).

The majority are Muslims in the country; therefore, they have strong Islamic values and ideologies in daily life guided by Al-Quran and Hadith (Prophetic teachings). Islamic belief plays a significant role in decision making, family dynamics, health practice and its service as well as utility among the believers (22). Moreover, Muslims are expected to seek treatments for curable sicknesses and to view incurable disease as God's will (23); some may view suffering as a way to atone for their past sins (24). Furthermore, a previous study shows that Indonesian students believe, obey, show gratitude, always follow Allah's commandment to avoid prohibitions as ways to maintain their health in contrast to Scandinavian, where spirituality and religious aspects regarding health practice is not a necessity (23). A study also concluded that spiritual intervention based on Islamic principles returns mental health and enhances hope and quality of life.

In conclusion, hospital management is expected to enhance nursing service quality by establishing sustainable education. This will improve nurses competencies and help escalate patient satisfaction as well as hospital service quality.
